# The Boy’s Venereal Peril

**Published:** 1903-10

**Authors:** 


THE BOY'S VENEREAL PERIL.—By F. C. V. Reprinted from the *Journal of the American Medical Association,* July 4, 1903. Published by press of A. M. A., 103 Dearborn Avenue, Chicago.

In this little pamphlet to the public, written unquestionably in a good cause, the author has wisely concealed his name and address as an evidence of his philanthropic motive. Alive to the dangers to which our American youth is subjected, uninstructed, he elaborates in this paper the dangers of self-abuse, the various venereal diseases, and warns against being "duped" by the various patent medicine advertisers.

Following the paper are impromptu discussions by prominent medical men. We read the paper with a good deal of pleasure, and are heartily in sympathy with the author. J. M. L.

